# Supplementary material for: Developing scandium and yttrium coordination chemistry to advance theranostic radiopharmaceuticals
Source: Commun Chem. 2020 May 15;3:61. doi: 10.1038/s42004-020-0307-0 (PMC9814396; doi:10.1038/s42004-020-0307-0)
Supplement: Supplementary file 1 — Supplementary Information [file 42004_2020_307_MOESM1_ESM.pdf]

Developing Scandium and Yttrium Coordination Chemistry to Advance Theranostic Radiopharmaceuticals

Korey P. Carter[a#], Gauthier J.-P. Deblonde[ab#], Trevor D. Lohrey[ac^], Tyler A. Bailey[ad], Dahlia D. An[a], Katherine M. Shield[ad], Wayne W. Lukens Jr.[a], and Rebecca J. Abergel*[ad]

[a] Chemical Sciences Division, Lawrence Berkeley National Laboratory, Berkeley, CA 94720, United States

[b] Glenn T. Seaborg Institute, Physical & Life Sciences, Lawrence Livermore National Laboratory, Livermore, California 94550, United States

[c] Department of Chemistry, University of California, Berkeley, CA 94720, United States

[d] Department of Nuclear Engineering, University of California, Berkeley, CA 94709, United States

[#] Authors contributed equally

[^] Present address: California Institute of Technology, 1200 E. California Blvd, MC 101-20, Pasadena, CA 91125, United States

E-mail: abergel@berkeley.edu

Supplementary Information

Supplementary Methods

Supplementary Figures and Tables

Supplementary References

Supplementary Methods

Caution! The PET isotope ^{86}Y ($t_{1/2}$=14.7 hours, 155.4 PBq/g) used in this work is highly radioactive. All work on ^{86}Y was done in facilities specially designed for the handling of transuranic and medical isotopes at the Lawrence Berkeley National Laboratory (LBNL).

Materials and Instrumentation

^{86}Y was supplied by the National Isotope Development Center (NIDC), which is part of United States Department of Energy Office of Science Isotope Program in the Office of Nuclear Physics. All chemicals were acquired commercially and used without further purification. The ligand 3,4,3-LI(1,2-HOPO), denoted 343-HOPO hereafter, was prepared by Ash Stevens, Inc. (Detroit, MI, USA) as previously described.[1] KCl (> 99.9995%, Sigma-Aldrich), 4-(2-hydroxyethyl)-1-piperazineethanesulfonic acid (hereafter called "HEPES") (99%, Sigma-Aldrich), potassium hydrogen phthalate (99.95%, Sigma-Aldrich), $EuCl_3 \cdot 6H_2O$ (> 99.9%, Sigma-Aldrich), $YCl_3 \cdot 6H_2O$ (99.99%, Sigma-Aldrich), and $ScCl_3 \cdot 6H_2O$ (99.999%, Sigma Aldrich) were used as received. Metal stock solutions were prepared by direct dissolution of the high purity salts in standard 0.1 M HCl solutions. Carbonate-free 0.1 M KOH was prepared from Baker *Dilut-It* concentrate and was standardized by titrating against 0.1 M potassium hydrogen phthalate. Standard solutions of 0.1 M HCl were purchased from VWR. All solutions were prepared using deionized water purified by a Millipore Milli-Q reverse osmosis cartridge system. Stocks were stored at 8°C in the dark between experiments. Static UV−Visible spectra were measured using a Cary 5G spectrophotometer using quartz cuvettes. ^{45}Sc NMR was recorded on a 300 MHz Bruker Ultrashield instrument at 25 ºC and all chemical shifts are reported as parts per million (ppm).

Titration Data Treatment

Spectrofluorimetric titration data sets were imported into the refinement program *HypSpec*[2] and analyzed by nonlinear least-squares refinement. All equilibrium constants were defined as cumulative formation constants, β_{mlh} according to Supplementary Equation 1, where the metal and ligand are designated as M and L, respectively.

$$m\mathrm{M} + h\mathrm{H} + l\mathrm{L} \rightleftharpoons [\mathrm{M}_m\mathrm{H}_h\mathrm{L}_l]; \qquad \beta_{mlh} = \frac{[\mathrm{M}_m\mathrm{H}_h\mathrm{L}_l]}{[\mathrm{M}]^m[\mathrm{H}]^h[\mathrm{L}]^l} \tag{1}$$

For species containing hydroxides, the h value is negative. The known stability constants listed in Supplementary Table 3 (i.e. ligand pKas and metal hydrolysis constants) were taken from the literature[3] and included in the refinement.

X-ray Crystallography

Single crystals suitable for X-ray diffraction of Sc(III)- and Y(III)-343-HOPO complexes were obtained by slow evaporation from 1:1 solutions in methanol and 0.5% w.t. DMF, with two equivalents of KCl included for charge balancing purposes. Crystals formed over one month at room temperature, and several crystals from each sample were transferred from their mother liquor, suspended in paratone oil, and inspected under a microscope equipped with a polarizing filter. Single crystals were isolated and mounted on MiTeGen micromounts and mounted crystals were immediately placed on the goniometer head of the diffractometer and cooled in a 100(2) K stream of dry nitrogen. Data collection was conducted at the Advanced Light Source station 12.2.1 at Lawrence Berkeley National Laboratory using a silicon-monochromated beam of 17 keV (λ = 0.7288 Å) synchrotron radiation. The Bruker APEX3 software package (including SAINT and SADABS)[4] was used throughout the data collection and reduction procedures, and

structures were solved and refined using SHELXT and SHELXL-2014, respectively,[5,6] in the WinGX software suite.[7] In both structures, all non-hydrogen atoms were located via difference Fourier maps and refined anisotropically when possible (several partially occupied water molecules in **1** were refined isotropically and without H-atoms), while hydrogen atoms were placed at their idealized positions and allowed to ride on the coordinates of their parent atoms. Significant disorder in the lattice solvent molecules of **1** was addressed using the SQUEEZE command in PLATON,[8] while disorder over two sites about the bound potassium cation was modeled via the PART command. Similar two-part disorder within part of the 343-HOPO ligand in **2** was also modeled satisfactorily via the PART command. Figures of the finalized structure were generated using Crystal Maker.[9] The crystallographic data and structure refinement details for complexes **1** and **2** are given in Supplementary Table 1. Thermal ellipsoid plots for both complexes are shown in Supplementary Figures 5 and 6.

Supplementary Table 1 Small molecule X-ray crystallographic data for complexes **1** and **2**.

	**1**	**2**
chem formula	$C_{37}H_{47.91}KN_9O_{16.45}Sc$	$C_{37}H_{39}KN_9O_{13}Y$
formula weight	966.07	947.80
crystal system	triclinic	triclinic
space group	P-1	P-1
a (Å)	10.2038(8)	10.1709(7)
b (Å)	14.6760(11)	11.0022(8)
c (Å)	18.5145(15)	17.6122(12)
α (deg)	76.724(3)	84.486(3)
β (deg)	75.801(3)	86.426(3)
γ (deg)	81.651(3)	86.669(3)
V (Å^3)	2604.4(4)	1955.3(2)
Z	2	2
T (K)	100(2)	100(2)
λ (Å), radiation type	0.72880, Synchotron	0.72880, Synchotron
D_{calc} (g cm^{-3})	1.232	1.610
μ (mm^{-1})	0.290	0.468
R_{int}	0.1155	0.0759
R1 [I>2σ(I)]	0.1003	0.0588
wR2 [I>2σ(I)]	0.3148	0.1434

PET Image Processing

Images were reconstructed using 3D Ordered Subset Expectation Maximum (OSEM3D) followed by Maximum a Posteriori (MAP) into a 128 x 128 x 63 (.85 x .85 x 1.2 mm^3) matrix. Image data underwent normalization to account for dead-time count losses, positron branching ratio, and physical decay to the time of injection. The counting rates in the processed images were converted to percentage-injected dose per gram of tissue (%ID/g) using a system calibration factor obtained by imaging a cylinder filled with ^{86}Y. The reported images are coronal maximum intensity projection (MIP) images. Images were analyzed using AMIDE.[10]

PET Resolution

Considering only the positron range and the acollinearity effect, the Ultimate Spatial Resolution can be defined according to Supplementary Equation 2, where s is the positron range and R is the detector ring radius.

$$\Gamma = \sqrt{s^2 + (0.0044R)^2} \quad (mm\, FWHM) \qquad (2)$$

Supplementary Table 2 displays the Ultimate Spatial Resolution for ^{44}Sc, ^{86}Y, and ^{68}Ga for both a pre-clinical scanner (assuming a radius of 10 mm) and a clinical scanner (assuming a radius of 400 mm). The positron range for ^{68}Ga was taken from Table 1 in the work of Moses,[11] and the positron range for both ^{44}Sc and ^{86}Y was linearly interpolated from the same table. Due to their shorter positron range, ^{44}Sc and ^{86}Y yield higher spatial resolution in PET images than ^{68}Ga.

Supplementary Table 2 Ultimate Spatial Resolution for ^{44}Sc, ^{86}Y, and ^{68}Ga for pre-clinical and clinical scanner.

Isotope	Mean β^+ Energy (keV)	Positron Range (FWHM, mm)	Ultimate Spatial Resolution for Pre-Clinical Scanners (FWHM, mm)	Ultimate Spatial Resolution for Clinical Scanners (FWHM, mm)
^{44}Sc	632.0	2.06	2.06	2.71
^{86}Y	652.1	2.17	2.17	2.79
^{68}Ga	836.0	2.83	2.83	3.33

Supplementary Figures and Tables

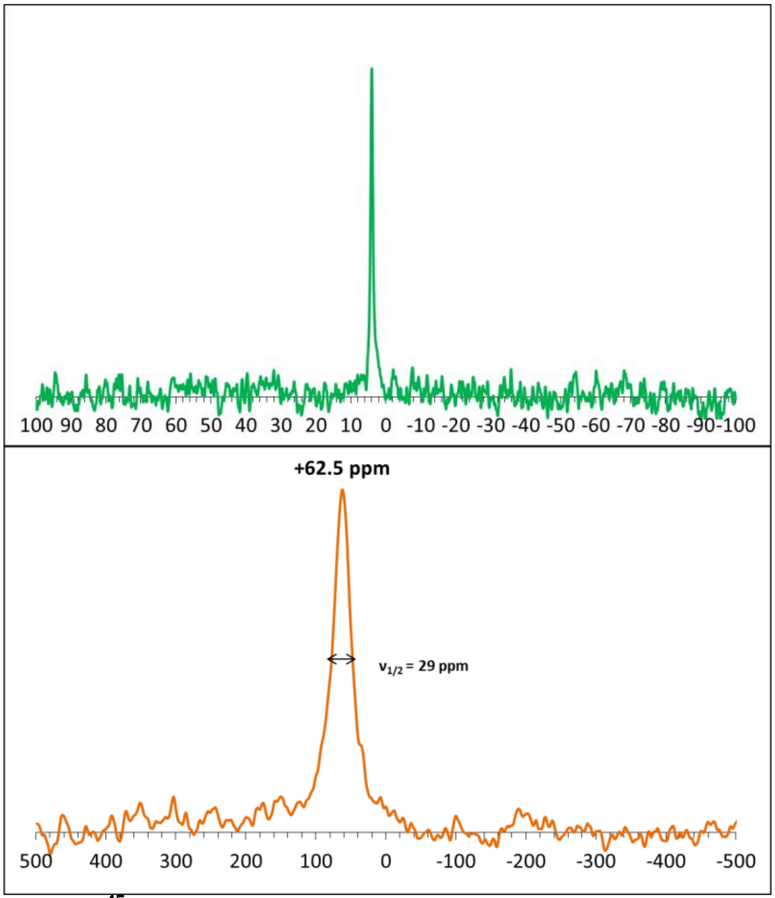

Supplementary Figure 1 ^{45}Sc NMR spectra. **Top**: $ScCl_3$ reference, 100 µM ScCl3 in 0.1 M HCl (NS = 43842). **Bottom**: 0.1 mM $[ScHOPO]^-$ in H_2O + 10 mM HEPES at pH 7.4 (NS = 2,872,037). T = 25°C.

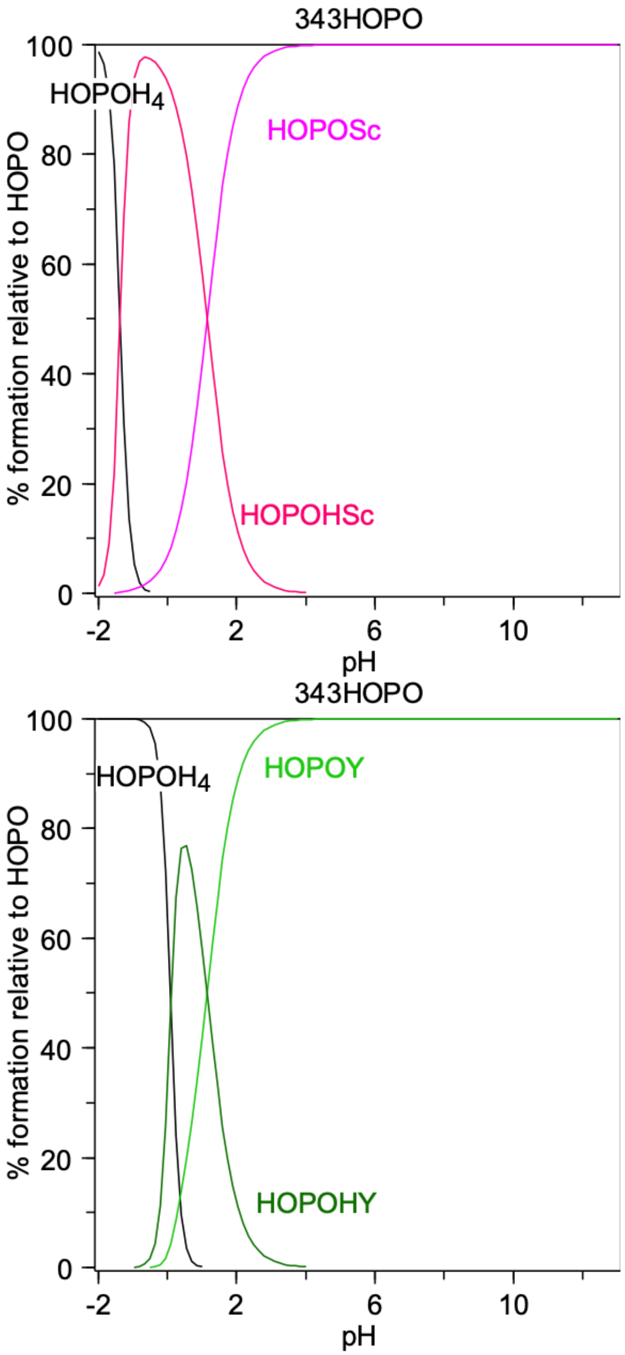

Supplementary Figure 2 Speciation diagrams for Sc^{3+} (**Top**) and Y^{3+} (**Bottom**) with 343-HOPO. The stability constants used for speciation calculations are included in Supplementary Table 3.

Supplementary Table 3 Proton independent stability constants used in the data refinement[3,12-15] or determined in this study. For [Sc-343-HOPOH] and [Y-343-HOPOH] species, log β values were extrapolated from the experimentally determined value for [Eu-343-HOPOH] (noted with * below).[16]

Species	*m, l, c, h*	log β	Reference
$[OH]^{-}$	0, 0, 0, -1	-13.76	12
$[HOPOH]^{3-}$	0, 1, 0, 1	6.64	3
$[HOPOH_2]^{2-}$	0, 1, 0, 2	12.32	3
$[HOPOH_3]^{-}$	0, 1, 0, 3	17.33	3
$[HOPOH_4]$	0, 1, 0, 4	21.20	3
$[ScOH]^{2-}$	1, 0, 0, -1	-4.8	13
$[Sc(OH)_2]^{-}$	1, 0, 0, -2	-10.2	13
$[Sc(OH)_3]$	1, 0, 0, -3	-16.1	13
$[YOH]^{2-}$	1, 0, 0, -1	-8.03	13
$[ScCl]^{2-}$	1, 0, 1, 0	1.95	14
$[ScCl_2]^{-}$	1, 0, 2, 0	3.5	14
$[YCl]^{2-}$	1, 0, 1, 0	-3.04	15
$[Sc\text{-}343\text{-}HOPO]^{-}$	1, 1, 0, 0	25.16 ± 0.01	this study
[Sc-343-HOPOH]	1, 1, 0, 1	26.3	this study*
$[Y\text{-}343\text{-}HOPO]^{-}$	1, 1, 0, 0	20.76 ± 0.09	this study
[Y-343-HOPOH]	1, 1, 0, 1	21.9	this study*

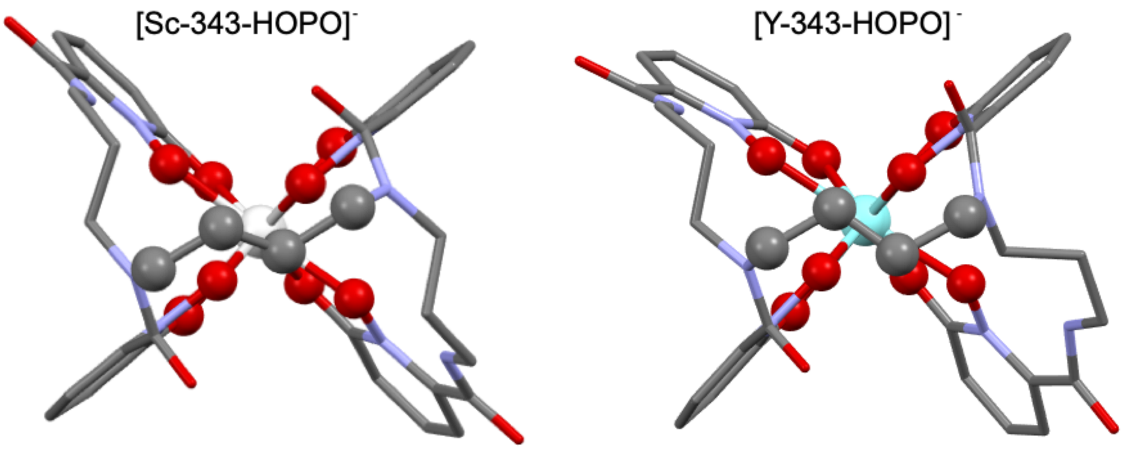

Supplementary Figure 3 Representation of crystal structures of Sc^{3+}- and Y^{3+}-343-HOPO complexes (**1** and **2**) highlighting racemic mixture of Δ(λ) and Λ(δ) conformations. The four-carbon chain of 343-HOPO (gray), the coordinating oxygen atoms (red), and the metal centers Sc^{3+} (white) and Y^{3+} (cyan) are displayed as spheres.

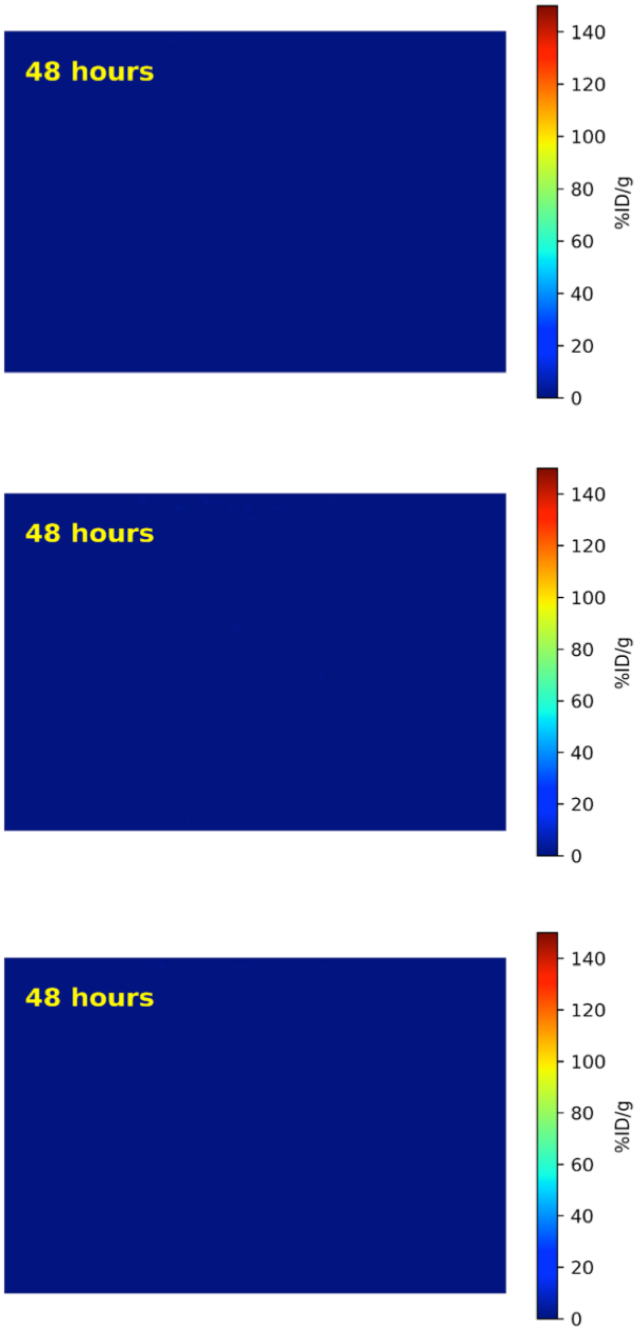

Supplementary Figure 4 Coronal PET images of ^{86}Y-343-HOPO. Three healthy mice were administered ^{86}Y-343-HOPO (93.1 µCi [3.44 MBq] in 10x PBS) via tail vein injection and imaged 48 hours after injection.

Supplementary Table 4 The percentage-injected dose per gram (%ID/g) for the gall bladder, gastrointestinal (GI) tract, and bladder. Uncertainties are reported as two sigma. The GI tract ROI was selected as this was region with the most accumulated activity. An asterisk (*) was used to denote organs that were not apparent at the specific timepoint. In these instances, a background ROI in the general location was reported.

**Mouse A1**	Gall Bladder (%ID/g)	GI Tract (%ID/g)	Bladder (%ID/g)
15 minutes	167.6 ± 100.7	117.2 ± 99.0	72.5 ± 19.6
2 hours	4.5 ± 4.1	99.7 ± 60.4	0.5 ± 0.6*
24 hours	0.01 ± 0.04*	2.7 ± 2.8	0.02 ± 0.05*
**Mouse A2**	Gall Bladder (%ID/g)	GI Tract (%ID/g)	Bladder (%ID/g)
15 minutes	152.1 ± 130.8	104.2 ± 100.8	35.8 ± 8.4
2 hours	79.4 ± 72.0	66.4 ± 37.6	0.4 ± 0.6*
24 hours	0.01 ± 0.04*	0.7 ± 1.1	0.01 ± 0.06*
**Mouse A3**	Gall Bladder (%ID/g)	GI Tract (%ID/g)	Bladder (%ID/g)
15 minutes	141.1 ± 97.4	144.6 ± 121.8	20.7 ± 4.4
2 hours	51.8 ± 40.6	84.3 ± 72.8	0.5 ± 0.5*
24 hours	0.05 ± 0.09*	0.5 ± 0.6	0.004 ± 0.03*

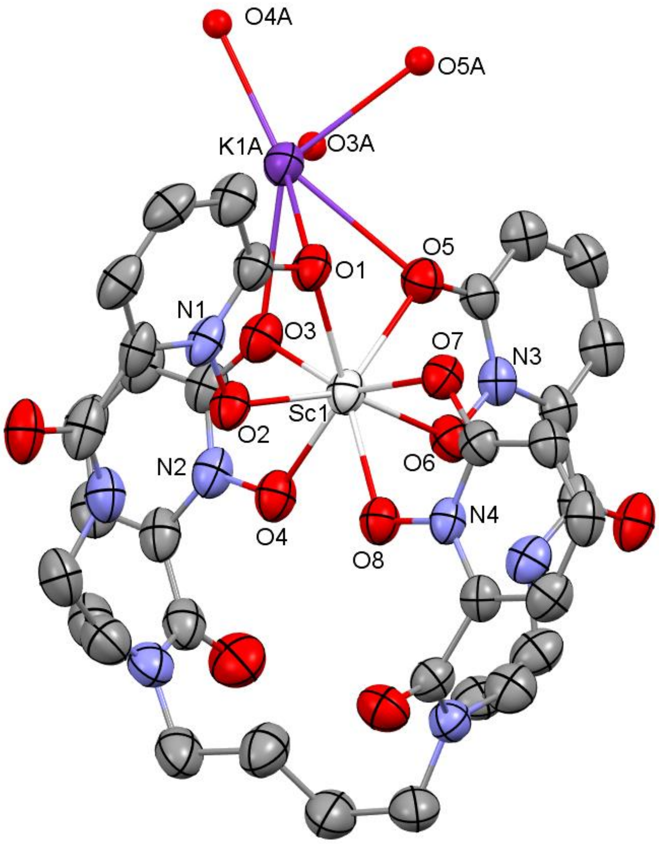

Supplementary Figure 5 Thermal ellipsoid plot of complex **1**. Ellipsoids are shown at 50% probability level. Several partial occupancy water molecules bound to the K^+ ion are displayed as spheres of a fixed radius. H-atoms and disordered sites of the 343-HOPO ligand and K^+ ion are omitted for clarity. Outer sphere molecules of DMF and water are also omitted for clarity.

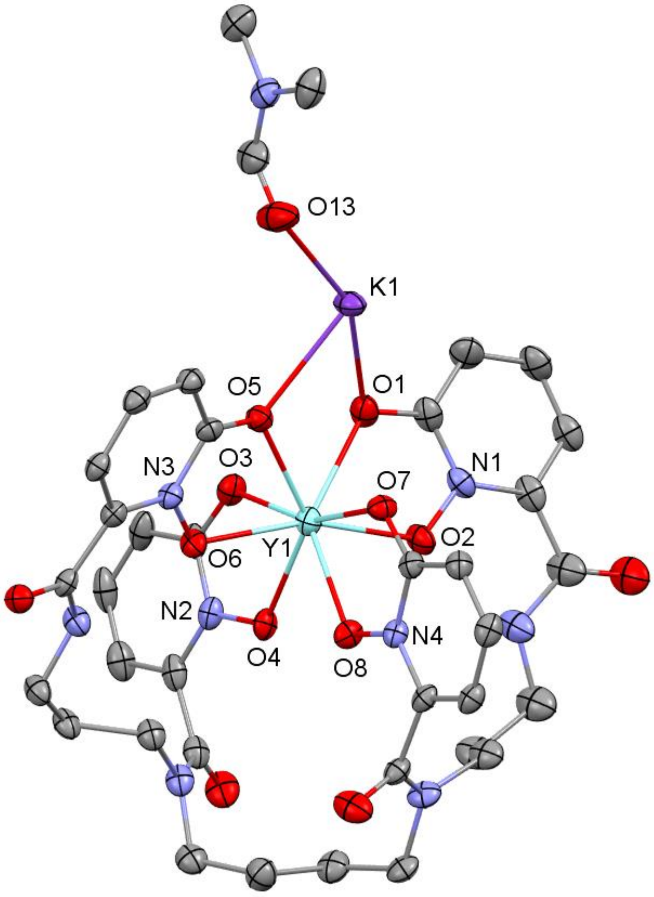

Supplementary Figure 6 Thermal ellipsoid plot of complex **2**. Ellipsoids are shown at 50% probability level. H-atoms and disordered sites of the 343-HOPO ligand are omitted for clarity.

Supplementary Table 5 RE-O bond lengths in complexes **1** and **2** with 343-HOPO.

**Complex (RE^{3+})**	$d_{RE1\text{-}O1}$ [Å]	$d_{RE1\text{-}O2}$ [Å]	$d_{RE1\text{-}O3}$ [Å]	$d_{RE1\text{-}O4}$ [Å]	$d_{RE1\text{-}O5}$ [Å]	$d_{RE1\text{-}O6}$ [Å]	$d_{RE1\text{-}O7}$ [Å]	$d_{RE1\text{-}O8}$ [Å]
**1 (Sc^{3+})**	2.255(4)	2.194(4)	2.207(4)	2.168(4)	2.273(4)	2.181(4)	2.192(4)	2.236(4)
**2 (Y^{3+})**	2.398(2)	2.326(2)	2.357(2)	2.331(2)	2.359(2)	2.343(2)	2.340(2)	2.320(2)

Supplementary References

1. Abergel, R. J. *et al.* Biomimetic Actinide Chelators: An Update on the Preclinical Development of the Orally Active Hydroxypyridonate Decorporation Agents 3,4,3-LI(1,2-HOPO) and 5-LIO(Me-3,2-HOPO). *Health Physics* **99**, 401-407, (2010).
2. Gans, P., Sabatini, A. & Vacca, A. Investigation of equilibria in solution. Determination of equilibrium constants with the HYPERQUAD suite of programs. *Talanta* **43**, 1739-1753, (1996).
3. Abergel, R. J., D'Aléo, A., Ng Pak Leung, C., Shuh, D. K. & Raymond, K. N. Using the Antenna Effect as a Spectroscopic Tool: Photophysics and Solution Thermodynamics of the Model Luminescent Hydroxypyridonate Complex $[Eu^{III}(3,4,3\text{-LI}(1,2\text{-HOPO}))]^{-}$. *Inorganic Chemistry* **48**, 10868-10870, (2009).
4. SADABS, APEX3, and SAINT (Bruker AXS Inc., Madison, Wisconsin, USA, 2015).
5. Sheldrick, G. SHELXT - Integrated space-group and crystal-structure determination. *Acta Crystallographica Section A* **71**, 3-8, (2015).
6. Sheldrick, G. Crystal structure refinement with SHELXL. *Acta Crystallographica Section C* **71**, 3-8, (2015).
7. Farrugia, L. WinGX and ORTEP for Windows: an update. *Journal of Applied Crystallography* **45**, 849-854, (2012).
8. Spek, A. PLATON SQUEEZE: a tool for the calculation of the disordered solvent contribution to the calculated structure factors. *Acta Crystallographica Section C* **71**, 9-18, (2015).
9. *CrystalMaker* v. 8.2.2 (Crystal Maker Software Limited, Bicester, England, 2009).
10. Loening, A. M. & Gambhir, S. S. AMIDE: A Free Software Tool for Multimodality Medical Image Analysis. *Molecular Imaging* **2**, 131-137, (2003).
11. Moses, W. W. Fundamental limits of spatial resolution in PET. *Nuclear Instruments and Methods in Physics Research Section A: Accelerators, Spectrometers, Detectors and Associated Equipment* **648**, S236-S240, (2011).
12. Martell, A. E. & Hancock, R. D. *Metal Complexes in Aqueous Solutions*. (Springer US, 1996).
13. Brown, P. L. & Ekberg, C. in *Hydrolysis of Metal Ions* Ch. 8, 225-324 (Wiley-VCH, 2016).
14. Melson, G. A. in *Scandium: Its Occurrence, Chemistry Physics, Metallurgy, Biology and Technology* (eds C. T. Horovitz *et al.*) Ch. 6, 111-138 (Academic Press, 1975).
15. Luo, Y.-R. & Byrne, R. H. Yttrium and Rare Earth Element Complexation by Chloride Ions at 25°C. *Journal of Solution Chemistry* **30**, 837-845, (2001).
16. Sturzbecher-Hoehne, M. *et al.* 3,4,3-LI(1,2-HOPO): In vitro formation of highly stable lanthanide complexes translates into efficacious in vivo europium decorporation. *Dalton Transactions* **40**, 8340-8346, (2011).
