# Supplementary material for: Developing scandium and yttrium coordination chemistry to advance theranostic radiopharmaceuticals
Source: Commun Chem. 2020 May 15;3:61. doi: 10.1038/s42004-020-0307-0 (PMC9814396; doi:10.1038/s42004-020-0307-0)
Supplement: Supplementary file 2 — Description of Additional Supplementary Files [file 42004_2020_307_MOESM2_ESM.pdf]

Description of Additional Supplementary Files

File Name: Supplementary Data 1
Description: crystallographic information file for complex 1

File Name: Supplementary Data 2
Description: crystallographic information file for complex 2
